# Association between commensality with depression and suicidal ideation of Korean adults: the sixth and seventh Korean National Health and Nutrition Examination Survey, 2013, 2015, 2017

**DOI:** 10.1186/s12937-020-00650-9

**Published:** 2020-12-02

**Authors:** Yoon Hee Son, Sarah Soyeon Oh, Sung-In Jang, Eun-Cheol Park, So-Hee Park

**Affiliations:** 1 College of Nursing, Ewha Womans University, Seoul, Republic of Korea; 2 Department of Public Health, Graduate School, Yonsei University, Seoul, Republic of Korea; 3 Department of Preventive Medicine and Institute of Health Services Research, Yonsei University College of Medicine, 50 Yonsei-ro, Seodaemun-gu, Seoul, 120-752 Republic of Korea

**Keywords:** Commensality, Eating alone, Depression, Suicidal ideation, Living alone, Region

## Abstract

**Objectives:**

This study investigated whether commensality (eating a meal with others) is associated with mental health (depression, suicidal ideation) in Korean adults over 19 years old.

**Methods:**

Our study employed data from the sixth and seventh Korea National Health and Nutritional Examination Surveys (KNHANES) for 2013, 2015, and 2017. The study population consisted of 14,125 Korean adults (5854 men and 8271 women). In this cross-sectional study, data were analyzed with the Rao-Scott chi-square test and multiple logistic regression to evaluate the association between commensality(0[includes skipping meals] to 3 times eating meals together) and both depression and suicidal ideation using select questions from the Mental Health Survey. By setting socioeconomic factors, health conditions, and behavioral factors as confounders, we conducted a subgroup analysis to reveal the effect on depression and suicidal ideation commensality.

**Results:**

Commensality was significantly associated with depression and suicidal ideation (*p* < 0.05). In both sexes, people who ate fewer meals together had poorer mental health. In a subgroup analysis, we revealed greater odds of developing depression in men when living in rural areas and belonging to low-income groups. In contrast, greater odds of suicidal ideation in men who ate alone when living in the city and belonging to high-income groups. On the other hand, Women in every region had greater odds of being depressed if they ate alone. And greater odds of suicidal ideation in women who ate alone when living in the city and belonging to medium-high income groups.

**Conclusions:**

Our analysis confirmed that Korean adults with lower chance of commensality had greater risk of developing depression and suicidal ideation. And it could be affected by individuals’ various backgrounds including socioeconomic status. As a result, to help people with depression and prevent a suicidal attempt, this study will be baseline research for social workers, educators and also policy developers to be aware of the importance of eating together.

## Highlights

- Commensality was significantly associated with depression and suicidal ideation.
- People who ate fewer meals together had poorer mental health.
- Men had greater odds of depression when living in rural areas and having low-income
- Women in every region had greater odds of being depressed if they ate alone

## Introduction

Mental illness affects 10% of the world’s population in modern society. Approximately 350 million people suffer from depression globally [1]. The causes of depression are various, including physiological factors, social psychological factors, environmental variation, and role changes as a family member or worker [2]. Depression deteriorates quality of life while leading to social problems (e.g., loss of support network or employment), increasing suicide risk [3, 4]. Indeed, suicide is a major clinical symptom of depression, highly correlated with suicidal ideation, and considerable effort has been devoted to examining the link between suicidal ideation and suicidal attempts [5].

South Korea currently has the highest suicide rate in the world at 25.6 per 100,000 people, with depression prevalence at 5.0% (men 3.0%, women 5.9%) (Statistics Korea, 2016). Mental-health problems are likely linked to a rapidly changing society with various demands at different ages, including marriage, childbirth, child rearing, employment, and retirement [6, 7]. Stress from sociological factors such as generational differences also contribute to mental health. As a coping mechanism, people may alter their behaviors, including eating habits. More research is needed on behavioral responses to mental stressors as they are expected to become increasingly common [8].

Commensality, or the act of eating meals together, has become an important health issue because eating alone appears to be associated with poorer mental health outcomes [9–14]. Although traditional customs emphasized commensality [15], people in modern societies are increasingly eating alone for various reasons. In particular, some people who dine alone have reported that they associate commensality with negative feelings because they do not have the freedom to eat what they like and are uncomfortable eating in the presence of others [16]. However, eating alone can exclude an individual from many positive effects of communal eating, including socializing and disclosure [17, 18].

The percentage of single-person households in South Korea has increased rapidly from 4.2% in 1975 to 28.6% in 2017, and this rise is projected to continue. For many Koreans, this recent decrease in number of family members occurs concurrently with eating alone involuntarily, leading to loneliness and social isolation [19]. Increasingly, work-related or personal problems are also causing modern young people to move their homes without settling down. Such changes mean the lack of opportunities to share their lives, including meals, with family or other close social partners, affecting physical health, cognition, emotional state, and behavior [20–22]. Most Korean adults either skip breakfast or eat the meal away from home. Additionally, some of them involuntarily spend lunchtime and dinnertime alone; the lack of meal-related social activities narrows their relationships and appears to generate depressive feelings [23]. Other studies in Korea likewise found that people who ate lunch or dinner alone were more depressed than those who ate commensally; these associations even stronger when eating alone was involuntary (caused by external situations) [8, 9, 11, 24]. Therefore, in this study, we examined recent data from South Korea to determine whether the association between commensality and mental health differs among subgroups and is affected by socio-economic factors such as age, household size, geographic regions, and household income level. Our findings should have important implications for developing appropriate measures to address depression and suicide.

## Materials and methods

### Study population and data

This study was conducted using the Korea National Health and Nutrition Examination Survey, which aims to provide data for the development and evaluation of health policy. The survey produces statistics regarding smoking, drinking, physical activity, and obesity for the World Health Organization and the Organization for Economic Cooperation and Development (OECD).

The survey was performed across 192 regions. Participants were selected through two-stage stratified cluster sampling step by step with regions and households. This study only used the first (2013) and third (2015) years of the sixth KNHANES, as well as the second (2017) year of the seventh KNHANES. These were the only years that included questions on suicidal ideation, suicidal plans, and suicidal attempts. Data from the three surveys were pooled during analysis.

Data from 3697 out of 18,341 adults (8088 men, 10,353 women) were excluded due to missing values in the household, health, and mental health surveys. The missing values on diagnosed depression were also excluded (519 participants). Although independent variables, depression and suicidal ideation, could be already affected by whether or not they are diagnosed depression, adults with diagnosed depression were included (643 participants) not to rule out the possibility that the commensality could actually have resulted in clinical depression. The final dataset for this study included 14,125 adults over 19 years old (5854 men and 8271 women).

### Measures

#### Outcome variables

Depression was assessed using one item on the mental health survey [25], “have you ever recently felt sad or desperate enough to experience negative effects in your everyday life for more than 2 weeks?”. Participants answered either “yes” or “no.” Based on these responses, they were categorized into two groups: (1) experienced depression, (2) did not experience depression.

Suicidal ideation was assessed instead of suicide directly owing to the difficulties of directly studying individuals who attempted or succeeded in suicide. Participants’ response to the question form the same survey, “have you ever seriously though of committing suicide within the last year?” was used to assess suicide ideation. Again, “yes” or “no” responses were used to categorized subjects into two groups: (1) experienced suicidal ideation, (2) did not experienced suicidal ideation. Since these data were obtained using a self-reported questionnaire and do not significantly represent clinical outcomes, those who were previously diagnosed with depression were not reclassified or treated differently.

#### Independent variable

Commensality was assessed using an item that asked whether participants ate each meal (breakfast, lunch, dinner) with family member or others within the past year. If a participant answered “yes” to “eating breakfast/lunch/dinner together,” then frequency of each meal was counted. The response “did not eat” (breakfast = 2918, lunch = 470, dinner = 291) was considered the same as “eating a meal alone,” because based on a previous study [26], we expected that skipping meals may also lead to lack of social exchange and elevate the risk of depression and suicidal ideation. Therefore, we re-classified eating habits into four groups: (1) eating no meal together, (2) eating one meal together, (3) eating two meals together, (4) eating all three meals together.

#### Covariates

The analysis examined a whole host of socioeconomic factors that could confound the relation between commensality and mental health, including gender, generation, household size, residential area, household income level, education level, and occupation. Chronic illness, smoking status, and drinking status were also included. Covariates were re-categorized based on previous research [12, 14, 20, 24, 26]: gender, generation age (20–29, 30–49, 50–64, ≥65), household size (alone, ≥1), residential area (metropolis [population over 1 million], city [population over 50,000], rural [population less than 50,000]), household income (low, medium-low, medium-high, high), completed education (≤elementary school, middle school, high school diploma, ≥bachelor’s degree), occupation (white collar, sales and services, blue collar, unemployed), presence of chronic illness (none, one, ≥2), smoking status (non-smoker, current smoker, past smoker), and drinking status (non-drinker, > 1 time per month, < 4 times per month, 2–3 times per week, ≥4 per week). Non-drinker group was analyzed as a reference, due to the nature of the questionnaires, to distinguish among non-drinker, drink less than once a month and drink once a month based on our previous study [27].

### Statistical analysis

Multiple logistic regression was performed to quantify the strength of associations between commensality and mental health variables through odd ratios (ORs) with 95% confidence intervals (CIs) and Rao-Scott chi-square tests. Individuals who ate three meals together were the reference category. We also conducted a subgroup analysis on depression and suicidal ideation among women and men separately to examine potential sex differences in the association with commensality. Marriage status as a variable with high multicollinearity (*P* ≥ 2) was excluded. All analyses were performed in SAS version 9.4 (SAS Institute, Cary, North Carolina, USA).

## Results

Of the study population, 2283 of 5854 men (39%) ate all three meals commensally, while 2724 of 8271 women (32.9%) had two meals commensally, as the highest percentage in their groups. Commensality was differentially associated with depression and suicidal ideation depending on socioeconomic or health characteristics (*p* < 0.05; Tables 1 and 2). Both mental health variables in men and women was significantly associated with household size, generation, household income, education, occupation, chronic illness, smoking status, and drinking status.

**Table 1:** General Characteristics of commensality and depression

														N (%)
	Depression													
	Men						*p*-value	Women						*p*-value
	Total		Yes		No			Total		Yes		No		
	N	(%)	N	(%)	N	(%)		N	(%)	N	(%)	N	(%)	
**Commensality**														
Eating 3 meals together	2283	39.0	172	31.7	2111	39.7	<.0001	2437	29.5	311	25.1	2126	30.2	<.0001
Eating 2 meals together	2062	35.2	158	29.2	1904	35.8		2724	32.9	350	28.2	2374	33.8	
Eating 1 meals together	904	15.4	89	16.4	815	15.3		1863	22.5	300	24.2	1563	22.2	
Eating no meals together	605	10.3	123	22.7	482	9.1		1247	15.1	280	22.6	967	13.8	
**Household member**														
Alone	584	10.0	115	21.2	469	8.8	<.0001	1036	12.5	233	18.8	803	11.4	<.0001
> 1	5270	90.0	427	78.8	4843	91.2		7235	87.5	1008	81.2	6227	88.6	
**Generation**														
20–29 years old	705	12.2	73	13.5	632	11.9	<.0001	828	10.0	128	10.3	700	10.0	<.0001
30–49 years old	1843	31.9	112	20.7	1731	32.6		2922	35.3	318	25.6	2604	37.0	
50–64 years old	1695	29.3	173	31.9	1522	28.7		2401	29.0	386	31.1	2016	28.7	
≥ 65 years old	1610	27.8	184	33.9	1427	26.9		2119	25.6	409	33.0	1710	24.3	
**Residential area**														
Metropolis	2498	42.7	236	43.5	2262	42.6	0.8737	3648	44.1	524	42.2	3124	44.4	0.0005
City	2256	38.5	208	38.4	2048	38.6		3213	38.8	458	36.9	2755	39.2	
Rural area	1100	18.8	98	18.1	1002	18.9		1410	17.0	259	20.9	1151	16.4	
**Household Income**														
Low	1105	19.1	186	34.3	919	17.3	<.0001	1737	21.0	433	34.9	1304	18.5	<.0001
Medium-low	1458	25.2	148	27.3	1310	24.7		2103	25.4	321	25.9	1782	25.3	
Medium-high	1577	27.3	94	17.3	1483	27.9		2177	26.3	265	21.4	1912	27.2	
High	1714	29.6	114	21.0	1600	30.1		2254	27.3	222	17.9	2032	28.9	
**Educational Attainment**														
Elementary School	1009	17.2	147	27.1	862	16.2	<.0001	2321	28.1	503	40.5	1818	25.9	<.0001
Middle School	648	11.1	70	12.9	578	10.9		843	10.2	146	11.8	697	9.9	
High School Diploma	2020	34.5	191	35.2	1829	34.4		2483	30.0	340	27.4	2143	30.5	
Bachelor’s Degree or Higher	2177	37.2	134	24.7	2043	38.5		2624	31.7	252	20.3	2372	33.7	
**Occupation**														
White Collar	1523	26.0	73	13.5	1450	27.3	<.0001	1660	20.1	145	11.7	1515	21.6	<.0001
Sales and Services	1015	17.3	90	16.6	925	17.4		1427	17.3	221	17.8	1206	17.2	
Blue Collar	1590	27.2	135	24.9	1455	27.4		1016	12.3	172	13.9	844	12.0	
Unemployed	1726	29.5	244	45.0	1482	27.9		4168	50.4	703	56.6	3465	49.3	
**Chronic Illnesses**														
None	3767	64.3	286	52.8	3481	65.5	<.0001	5171	62.5	655	52.8	4516	64.2	<.0001
1	1180	20.2	137	25.3	1043	19.6		1519	18.4	261	21.0	1258	17.9	
2 or more	907	15.5	119	22.0	788	14.8		1581	19.1	325	26.2	1256	17.9	
**Smoking**														
Current Smoker	2013	34.4	210	38.7	1803	33.9	0.0444	384	4.6	110	8.9	274	3.9	<.0001
Past Smoker	2518	43.0	227	41.9	2291	43.1		443	5.4	83	6.7	360	5.1	
Non-Smoker	1323	22.6	105	19.4	1218	22.9		7444	91.1	1048	84.4	6396	91.0	
**Drinking**														
Non-drinker	290	5.0	44	8.1	246	4.6	<.0001	1518	18.6	261	21.0	1257	17.9	<.0001
< 1 time per/month	1440	24.6	149	27.5	1291	24.3		3493	42.8	517	41.7	2976	42.3	
< 4 times per/month	2045	34.9	148	27.3	1897	35.7		2439	29.9	323	26.0	2116	30.1	
2–3 times per week	1373	23.5	112	20.7	1261	23.7		629	7.7	94	7.6	535	7.6	
≥ 4 per week	706	12.1	89	16.4	617	11.6		192	2.4	46	3.7	146	2.1	
**Total**	5854	100.0	542	9.3	5312	90.7		8271	100.0	1241	15.0	7030	85.0

**Table 2:** General Characteristics of commensality and suicidal ideation

														N (%)
	Suicidal ideation													
	Men						*p*-value	Women						*p*-value
	Total		Yes		No			Total		Yes		No		
	N	(%)	N	(%)	N	(%)		N	(%)	N	(%)	N	(%)	
**Commensality**														
Eating 3 meals together	2283	39.0	73	26.9	2210	39.6	<.0001	2437	29.5	108	22.5	2329	29.9	<.0001
Eating 2 meals together	2062	35.2	66	24.4	1996	35.8		2724	32.9	117	24.4	2607	33.5	
Eating 1 meals together	904	15.4	53	19.6	851	15.2		1863	22.5	128	26.7	1735	22.3	
Eating no meals together	605	10.3	79	29.2	526	9.4		1247	15.1	126	26.3	1121	14.4	
**Household member**														
Alone	584	10.0	75	27.7	509	9.1	<.0001	1036	12.5	101	21.1	935	12.0	<.0001
> 1	5270	90.0	196	72.3	5074	90.9		7235	87.5	378	78.9	6857	88.0	
**Generation**														
20–29 years old	705	12.2	26	9.6	679	12.2	<.0001	828	10.0	46	9.6	782	10.0	<.0001
30–49 years old	1843	31.9	49	18.1	1794	32.1		2922	35.3	118	24.6	2804	36.0	
50–64 years old	1695	29.3	87	32.1	1608	28.8		2401	29.0	145	30.3	2257	29.0	
≥ 65 years old	1610	27.8	109	40.2	1502	26.9		2119	25.6	170	35.5	1949	25.0	
**Residential area**														
Metropolis	2498	42.7	114	42.1	2384	42.7	0.7140	3648	44.1	197	41.1	3451	44.3	0.0269
City	2256	38.5	101	37.3	2155	38.6		3213	38.8	179	37.4	3034	38.9	
Rural area	1100	18.8	56	20.7	1044	18.7		1410	17.0	103	21.5	1307	16.8	
**Household Income**														
Low	1105	19.1	125	46.1	980	17.6	<.0001	1737	21.0	185	38.6	1552	19.9	<.0001
Medium-low	1458	25.2	67	24.7	1391	24.9		2103	25.4	133	27.8	1970	25.3	
Medium-high	1577	27.3	34	12.5	1543	27.6		2177	26.3	90	18.8	2087	26.8	
High	1714	29.6	45	16.6	1669	29.9		2254	27.3	71	14.8	2183	28.0	
**Educational Attainment**														
Elementary School	1009	17.2	86	31.7	923	16.5	<.0001	2321	28.4	215	44.9	2106	27.0	<.0001
Middle School	648	11.1	49	18.1	599	10.7		843	10.3	46	9.6	797	10.2	
High School Diploma	2020	34.5	86	31.7	1934	34.6		2483	30.4	144	30.1	2339	30.0	
Bachelor’s Degree or Higher	2177	37.2	50	18.5	2127	38.1		2624	32.1	74	15.4	2550	32.7	
**Occupation**														
White Collar	1523	26.0	30	11.1	1493	26.7	<.0001	1660	20.1	51	10.6	1609	20.6	<.0001
Sales and Services	1015	17.3	32	11.8	983	17.6		1427	17.3	84	17.5	1343	17.2	
Blue Collar	1590	27.2	65	24.0	1525	27.3		1016	12.3	58	12.1	958	12.3	
Unemployed	1726	29.5	144	53.1	1582	28.3		4168	50.4	286	59.7	3882	49.8	
**Chronic Illnesses**														
None	3767	64.3	128	47.2	3639	65.2	<.0001	5171	62.5	237	49.5	4934	63.3	<.0001
1	1180	20.2	78	28.8	1102	19.7		1519	18.4	112	23.4	1407	18.1	
2 or more	907	15.5	65	24.0	842	15.1		1581	19.1	130	27.1	1451	18.6	
**Smoking**														
Current Smoker	2013	34.4	114	42.1	1899	34.0	0.0049	384	4.6	60	12.5	324	4.2	<.0001
Past Smoker	2518	43.0	114	42.1	2404	43.1		443	5.4	41	8.6	402	5.2	
Non-Smoker	1323	22.6	43	15.9	1280	22.9		7444	91.1	378	78.9	7066	90.7	
**Drinking**														
Non-drinker	290	5.0	20	7.4	270	4.8	<.0001	1518	18.6	101	21.1	1417	18.2	<.0001
< 1 time per/month	1440	24.6	91	33.6	1349	24.2		3493	42.8	199	41.5	3294	42.3	
< 4 times per/month	2045	34.9	68	25.1	1977	35.4		2439	29.9	115	24.0	2324	29.8	
2–3 times per week	1373	23.5	39	14.4	1334	23.9		629	7.7	37	7.7	592	7.6	
≥ 4 per week	706	12.1	53	19.6	653	11.7		192	2.4	27	5.6	165	2.1	
**Total**	5854		271	4.6	5583	95.4		8271		479	5.8	7792	94.2	

### Associations between commensality and depression

**Fig. 1:**
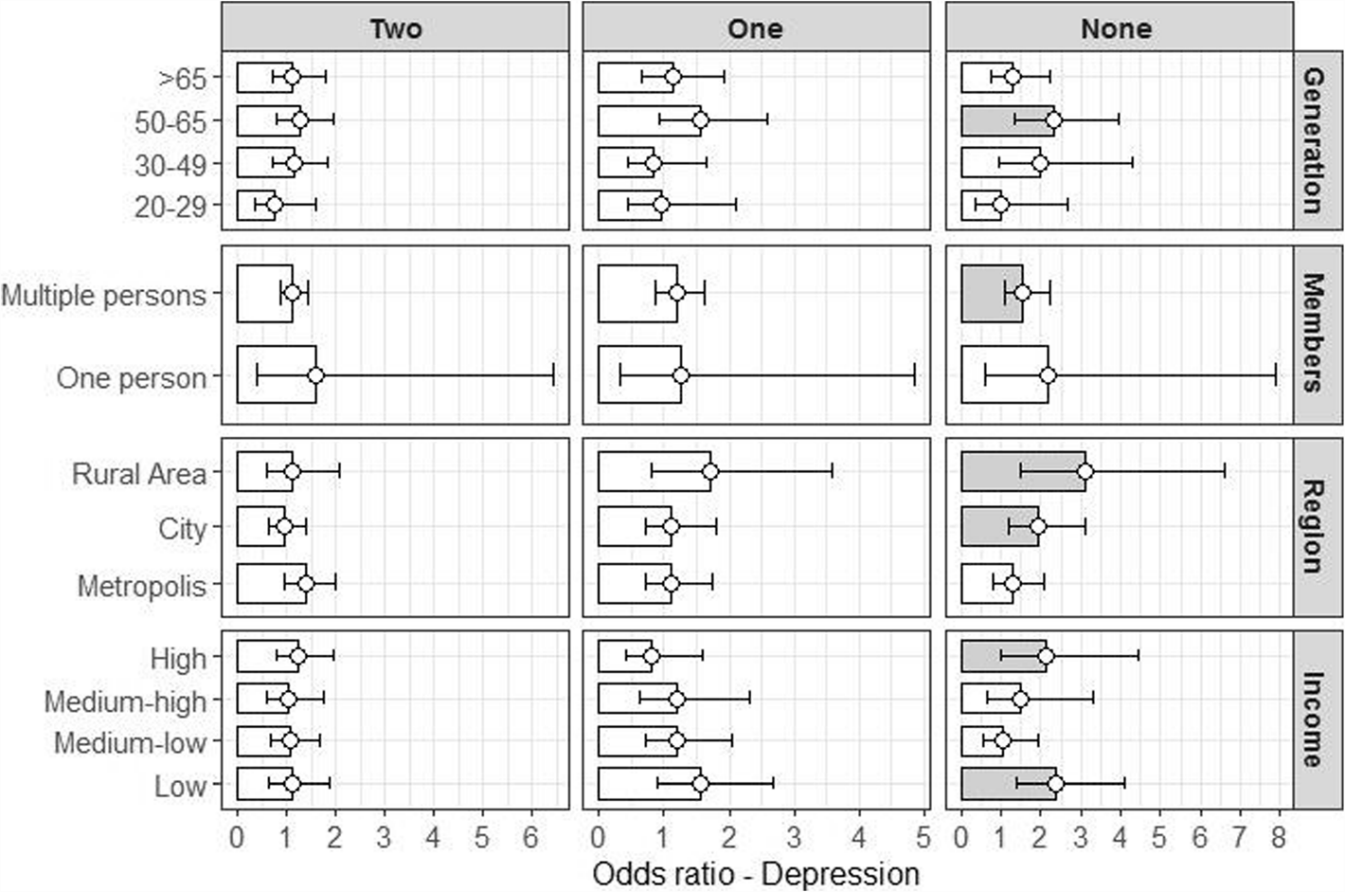
Commensality and depression: generation, household members, region, household income

Relative to those who had all three meals together, men who ate every meal alone were up to 1.72 times (OR: 1.72, 95% CI: 1.27–2.34) more likely to be depressed, while women who ate alone were 1.58 times (OR: 1.58, 95% CI: 1.28–1.95) more likely to be depressed. There was a weaker association between depression and commensality among the ≥65 years old category than the 20–29 year old category (reference group) for both men (OR: 0.54, 95% CI: 0.37–0.80) and women (OR: 0.49, 95% CI: 0.35–0.68). Men who lived with others had a significantly greater association between commensality and depression (OR: 1.65, 95% CI: 0.37–0.80) than those who lived alone (Table 3). The result of associations between commensality and depression was shown in Fig. 1.

**Table 3:** Association between commensality and general characteristics of depression

	Depression			
	Men (***n*** = 5854)		Women (***n***-8271)	
	Odds Ratio	95% CI^a^	Odds Ratio	95% CI
**Commensality**^**b**^				
Eating 3 meals together	1.00	–	1.00	**–**
Eating 2 meals together	1.15	(0.90**–**1.46)	1.15	(0.97**–**1.36)
Eating 1 meals together	1.17	(0.87**–**1.56)	**1.36**	**(1.13–1.63)**
Eating no meals together	**1.72**	**(1.27–2.34)**	**1.58**	**(1.28–1.95)**
**Household member**				
Alone	1.00	–	1.00	**–**
> 1	**1.61**	**(1.22–2.14)**	**0.90**	(0.73**–**1.01)
**Generation**				
20–29 years old	1.00	–	1.00	**–**
30–49 years old	0.72	(0.59**–**1.01)	**0.66**	**(0.52–0.83)**
50–64 years old	0.80	(0.55**–**1.15)	**0.70**	**(0.53–0.91)**
≥ 65 years old	**0.54**	**(0.37–0.80)**	**0.49**	**(0.35–0.68)**
**Residential area**				
Metropolis	1.00	–	1.00	**–**
City	0.97	(0.79–1.19)	0.99	(0.86**–**1.14)
Rural area	0.82	(0.63–1.07)	1.17	(0.98**–**1.39)
**Household Income**				
Low	1.00	–	1.00	**–**
Medium-low	0.80	(0.61**–**1.03)	**0.62**	**(0.52–0.74)**
Medium-high	**0.51**	**(0.37–0.69)**	**0.53**	**(0.44–0.65)**
High	**0.63**	**(0.47–0.85)**	**0.48**	**(0.38–0.59)**
**Educational Attainment**				
Elementary School	1.00	–	1.00	**–**
Middle School	0.85	(0.62–1.17)	0.85	(0.68**–**1.07)
High School Diploma	0.82	(0.62**–**1.10)	**0.69**	**(0.56–0.85)**
Bachelor’s Degree or Higher	**0.70**	**(0.50–0.98)**	**0.56**	**(0.42–0.71)**
**Occupation**				
White Collar	1.00	–	1.00	**–**
Sales and Services	**1.49**	**(1.05–2.13)**	1.23	(0.96**–**1.58)
Blue Collar	1.34	(0.95–1.89)	1.29	(0.98**–**1.69)
Unemployed	**1.92**	**(1.37–2.69)**	**1.36**	**(1.10–1.69)**
**Chronic Illnesses**				
None	1.00	–	1.00	**–**
1	**1.38**	**(1.08–1.78)**	1.10	(0.91**–**1.33)
2 or more	**1.43**	**(1.10–1.87)**	**1.26**	**(1.04–1.53)**
**Smoking**				
Non-smoker	1.00	–	1.00	**–**
Current smoker	1.24	(0.95**–**1.62)	**2.15**	**(1.67–2.76)**
Past smoker	1.06	(0.81**–**1.38)	**1.30**	**(1.00–1.69)**
**Drinking**				
Non-drinker	1.00	–	1.00	**–**
< 1 time per/month	0.70	(0.48**–**1.02)	1.02	(0.86**–**1.22)
< 4 times per/month	**0.58**	**(0.39–0.84)**	0.99	(0.81**–**1.20)
2–3 times per week	**0.63**	**(0.42–0.93)**	1.06	(0.80**–**1.40)
≥ 4 per week	0.83	(0.55–1.25)	1.43	(0.96**–**2.13)

^a^*CI* Confidence Interval

^b^Commensality is analyzed by Controlled variables includes household members, generation, Residential area, household income, educational attainment, occupation, chronic illnesses, smoking, drinking

### Associations between commensality and suicidal ideation

**Fig. 2:**
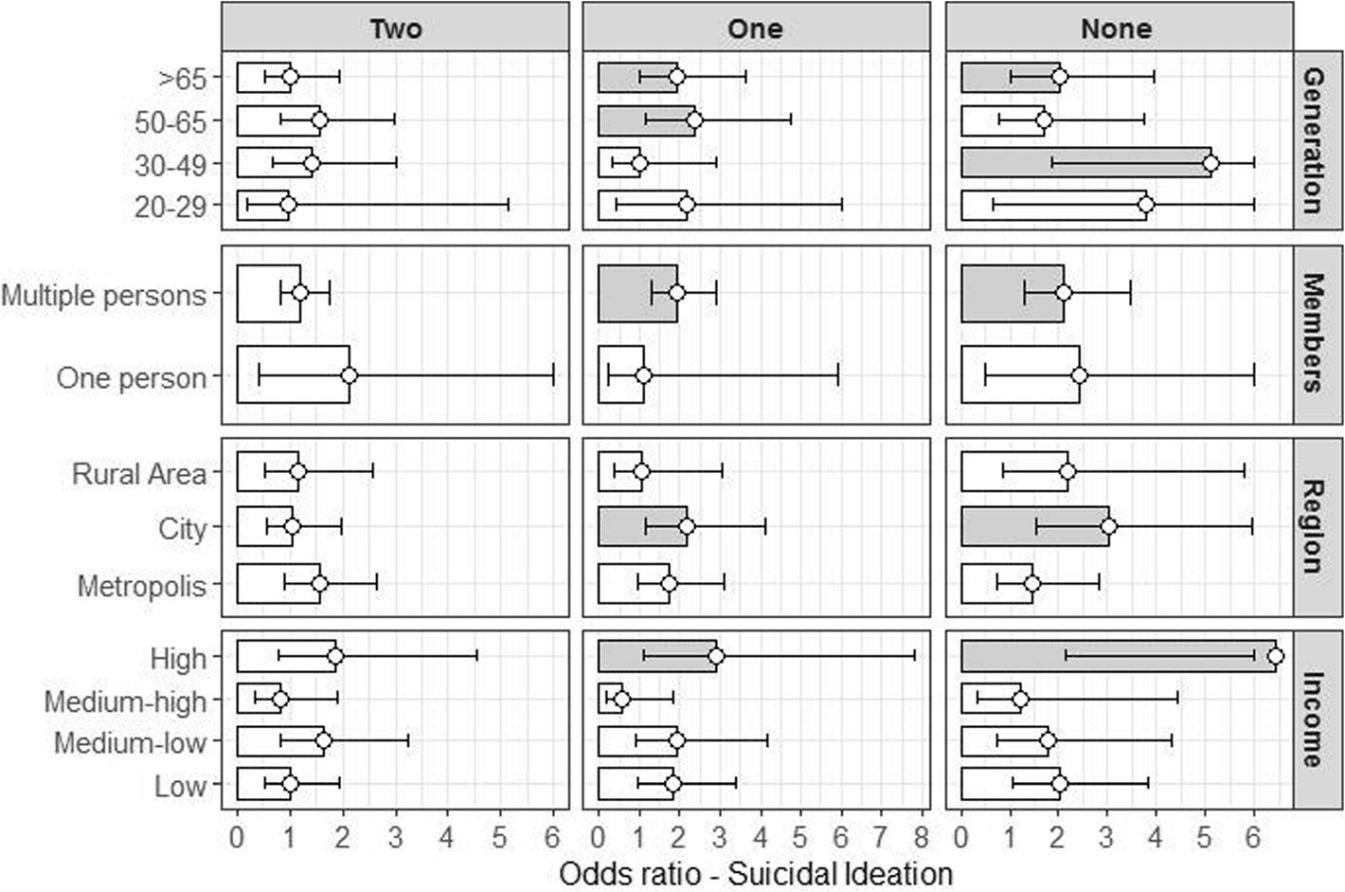
Commensality and suicidal ideation: generation, household members, region, household income

Men eating one meal together (OR: 1.77, 95% CI: 1.19–2.62), men eating all meals alone (OR: 2.16, 95% CI: 1.41–3.30), women eating one meal together (OR 1.64, 95% CI: 1.24–2.17), and women eating all meals alone (OR: 1.94, 95% CI: 1.41–2.67) were all highly associated with suicidal ideation. Suicidal ideation in men who lived with others (household size > 1) was also more likely to be associated with commensality than those who lived alone (OR: 1.61, 95% CI: 1.10–2.37). Women in the 20–29 age group experienced a stronger association between suicidal ideation and commensality than other generations. In addition, this association was stronger among women who lived in rural regions (OR 1.02, 95% CI: 0.82–1.26) or cities (OR 1.18, 95% CI: 0.90–1.54) compared with those living in metropolitan areas, although the difference was not significant (Table 4). The result of associations between commensality and suicidal ideation was shown in Fig. 2.

**Table 4:** Association between commensality and general characteristics of suicidal ideation

	Suicidal ideation			
	Men (***n*** = 5854)		Women (***n*** = 8271)	
	Odds Ratio	95% CI^a^	Odds Ratio	95% CI
**Commensality**^b^				
Eating 3 meals together	1.00	–	1.00	**–**
Eating 2 meals together	1.28	(0.90–1.82)	1.09	(0.82**–**1.45)
Eating 1 meals together	**1.77**	**(1.19–2.62)**	**1.64**	**(1.24–2.17)**
Eating no meals together	**2.16**	**(1.41–3.30)**	**1.94**	**(1.41–2.67)**
**Household member**				
Alone	1.00	–	1.00	**–**
> 1	**1.61**	**(1.10–2.37)**	0.81	(0.59**–**1.12)
**Generation**				
20–29 years old	1.00	–	1.00	**–**
30–49 years old	1.03	(0.60–1.76)	0.74	(0.52**–**1.06)
50–64 years old	1.04	(0.59–1.83)	0.74	(0.48**–**1.14)
≥ 65 years old	0.78	(0.44**–**1.37)	**0.52**	**(0.31–0.88)**
**Residential area**				
Metropolis	1.00	–	1.00	**–**
City	0.99	(0.74–1.32)	1.02	(0.82**–**1.26)
Rural area	1.03	(0.72–1.46)	1.18	(0.90**–**1.54)
**Household Income**				
Low	1.00	–	1.00	**–**
Medium-low	**0.59**	**(0.42–0.83)**	**0.68**	**(0.52–0.90)**
Medium-high	**0.33**	**(0.22–0.51)**	**0.49**	**(0.36–0.66)**
High	**0.47**	**(0.31–0.72)**	**0.44**	**(0.31–0.62)**
**Educational Attainment**				
Elementary School	1.00	–	1.00	**–**
Middle School	1.22	(0.82**–**1.81)	**0.64**	**(0.45–0.91)**
High School Diploma	0.82	(0.56**–**1.20)	0.74	(0.52**–**1.03)
Bachelor’s Degree or Higher	**0.58**	**(0.36–0.94)**	**0.41**	**(0.26–0.63)**
**Occupation**				
White Collar	1.00	–	1.00	**–**
Sales and Services	0.90	(0.53–1.55)	1.04	(0.70**–**1.55)
Blue Collar	1.13	(0.69–1.84)	0.91	(0.59**–**1.40)
Unemployed	**1.77**	**(1.09–2.85)**	1.21	(0.86**–**1.71)
**Chronic Illnesses**				
None	1.00	**–**	1.00	**–**
1	**1.47**	**(1.04–2.06)**	1.26	(0.95**–**1.67)
2 or more	1.34	(0.93–1.92)	1.27	(0.94**–**1.72)
**Smoking**				
Non-smoker	1.00	–	1.00	**–**
Current smoker	**1.50**	**(1.01–2.22)**	**2.85**	**(2.05–3.97)**
Past smoker	1.10	(0.75–1.63)	**1.70**	**(1.19–2.44)**
**Drinking**				
Non-drinker	1.00	–	1.00	**–**
< 1 time per/month	1.08	(0.65**–**1.79)	1.04	(0.80**–**1.36)
< 4 times per/month	0.77	(0.45**–**1.31)	0.95	(0.70**–**1.29)
2–3 times per week	0.58	(0.33**–**1.02)	1.04	(0.67**–**1.63)
≥ 4 per week	1.22	(0.70**–**2.13)	**1.98**	**(1.19–3.30)**

^a^*CI* Confidence Interval

l

^b^Commensality is analyzed by Controlled variables includes household members, generation, Residential area, household income, educational attainment, occupation, chronic illnesses, smoking, drinking

### Subgroup of depression and suicidal ideation among men

Subgroup analysis showed that in men of the 50–64 age group, depression was significantly associated with eating all meals alone (OR: 2.32, 95% CI: 1.35–3.97). Looking within multi-person households, depression was significantly associated with eating alone (OR: 1.55, 95% CI: 1.08–2.24). Within residential area, eating fewer meals together meant being 1.92 times more likely to be depressed when living in cities (OR:1.92, 95% CI 1.18–3.12) and 3.11 times more likely in rural areas (OR: 3.11, 95% CI: 1.47–6.60). Similarly, suicidal ideation was significantly associated with eating fewer meals together among men. Within generations, the 30–49 age group had the highest association between eating all meals alone and suicidal ideation (OR: 5.11, 95% CI: 1.87–14.00). Those who lived in cities were more likely to have an association between eating no meals commensally and suicidal ideation (OR: 3.10, 95% CI: 1.53–5.94). Men in the high-income group were significantly more likely to have suicidal ideation if they ate only one meal together (OR: 2.92, 95% CI: 1.09–7.82) or ate all meals alone (OR: 6.45, 95% CI: 2.15–19.33) (Table 5).

**Table 5:** Association between commensality and depression and suicidal ideation in subgroups: Men

**Depression (*n*** **= 542)**	**Commensality (Number of meals together)**^**b**^							
	**Case(n)**	**3(*n*** **= 172)**	**2(*n*** **= 158)**		**1(*n*** **= 89)**		**None(*n*** **= 123)**	
		Odds Ratio	Odds Ratio	95% CI^a^	Odds Ratio	95% CI^a^	Odds Ratio	95% CI
Generation								
20–29 years old	71	1.00	0.77	(0.37–1.60)	0.96	(0.44–2.09)	0.99	(0.37**–**2.65)
30–49 years old	112	1.00	1.14	(0.71–1.82)	0.84	(0.43–1.65)	2.00	(0.93–4.30)
50–64 years old	173	1.00	1.26	(0.81–1.94)	1.56	(0.94–2.59)	**2.32**	**(1.35–3.97)**
≥ 65 years old	184	1.00	1.13	(0.71–1.79)	1.13	(0.66–1.92)	1.29	(0.74–2.25)
Household members								
One person	115	1.00	1.59	(0.39**–**6.43)	1.25	(0.32–4.86)	2.17	(0.60**–**7.90)
Multiple persons(≥1)	427	1.00	1.12	(0.88**–**1.43)	1.20	(0.88–1.62)	**1.55**	**(1.08–2.24)**
Region								
Metropolis	236	1.00	1.40	(0.97–2.01)	1.12	(0.73**–**1.73)	1.28	(0.79–2.06)
City	208	1.00	0.94	(0.64–1.38)	1.12	(0.71**–**1.79)	**1.92**	**(1.18–3.12)**
Rural area	98	1.00	1.13	(0.61–2.09)	1.71	(0.81–3.57)	**3.11**	**(1.47–6.60)**
Household Income								
Low	186	1.00	1.10	(0.65–1.86)	1.56	(0.90–2.69)	**2.39**	**(1.40–4.10)**
Medium-low	148	1.00	1.07	(0.68–1.67)	1.21	(0.72–2.03)	1.03	(0.55–1.93)
Medium-high	94	1.00	1.04	(0.61–1.77)	1.20	(0.63–2.31)	1.49	(0.67–3.32)
High	114	1.00	1.24	(0.78–1.97)	0.81	(0.41–1.59)	**2.14**	**(1.02–4.46)**
**Suicidal ideation (*n*** **= 271)**	**Case(n)**	**3(*n*** **= 77)**	**2(*n*** **= 66)**		**1(*n*** **= 53)**		**None(*n*** **= 79)**	
		Odds Ratio	Odds Ratio	95% CI^a^	Odds Ratio	95% CI^a^	Odds Ratio	95% CI
Generation								
20–29 years old	26	1.00	0.97	(0.18–5.14)	2.16	(0.43–10.96)	3.77	(0.65–21.89)
30–49 years old	49	1.00	1.41	(0.65**–**3.03)	1.00	(0.34**–**2.92)	**5.11**	**(1.87–14.00)**
50–64 years old	87	1.00	1.56	(0.82**–**2.99)	**2.37**	**(1.17–4.75)**	1.71	(0.77–3.75)
≥ 65 years old	109	1.00	1.01	(0.52–1.95)	**1.92**	**(1.02–3.61)**	**2.01**	**(1.02–3.95)**
Household members								
One person	75	1.00	2.14	(0.40–11.46)	1.11	(0.21–5.92)	2.43	(0.51–11.53)
Multiple persons(≥1)	196	1.00	1.19	(0.81–1.74)	**1.94**	**(1.28–2.92)**	**2.11**	**(1.29–3.46)**
Region								
Metropolis	115	1.00	1.55	(0.91**–**2.66)	1.72	(0.95**–**3.09)	1.44	(0.73–2.82)
City	101	1.00	1.04	(0.55**–**1.96)	**2.15**	**(1.13–4.11)**	**3.01**	**(1.53–5.94)**
Rural area	56	1.00	1.15	(0.51–2.57)	1.03	(0.35–3.05)	2.20	(0.84–5.79)
Household Income								
Low	125	1.00	0.99	(0.51**–**1.92)	1.83	(0.98–3.40)	**2.02**	**(1.07–3.83)**
Medium-low	67	1.00	1.65	(0.83**–**3.25)	1.95	(0.92–4.15)	1.78	(0.74–4.29)
Medium-high	34	1.00	0.81	(0.35**–**1.91)	0.58	(0.18–1.83)	1.23	(0.34–4.41)
High	45	1.00	1.87	(0.77–4.53)	**2.92**	**(1.09–7.82)**	**6.45**	**(2.15–19.33)**

^a^*CI* Confidence Interval

^c^Commensality is analyzed by Controlled variables includes household members, generation, Residential area, household income, educational attainment, occupation, chronic illnesses, smoking, drinking, except each subgroup variable

### Subgroup of depression and suicidal ideation among women

Women who were ≥ 65 years old were 1.72 times more likely to have depression if they only ate one meal commensally (OR: 1.72, 95% CI: 1.21–2.45) and 3.04 times more likely if they ate alone (OR: 2.04, 95% CI: 1.44–2.89). Similar to men, women who lived in multi-person households were 1.38 times more likely to have depression if they ate one meal together (OR: 1.38, 95% CI: 1.14–1.67) and 1.56 times more likely if they ate entirely alone (OR: 1.56, 95% CI: 1.23–1.99). Women in every region had greater odds of being depressed if they ate alone (metropolitan area, OR: 1.70, 95% CI: 1.23–2.34; city, OR: 1.73, 95% CI: 1.22–2.46; rural area, OR: 1.21, 95% CI: 0.73–2.01). Among medium-high income women, eating two meals together (OR: 1.76, 95% CI: 1.22–2.55), eating one meal together (OR: 2.02, 95% CI: 1.37–2.97), eating no meals together (OR: 2.04, 95% CI: 1.24–3.35) all increased the odds of being depressed (Table 6).

**Table 6:** Association between commensality and depression and suicidal ideation in subgroups: Women

**Depression (*n*** **= 1241)**	**Case(n)**	**Commensality (Number of meals together)**^**b**^						
		**3(*n*** **= 311)**	**2(*n*** **= 350)**		**1(*n*** **= 300)**		**None(*n*** **= 280)**	
		Odds Ratio	Odds Ratio	95% CI^a^	Odds Ratio	95% CI^a^	Odds Ratio	95% CI
Generation								
20–29 years old	128	1.00	0.95	(0.54**–**1.66)	1.21	(0.68–2.17)	1.15	(0.54–2.44)
30–49 years old	318	1.00	1.18	(0.86**–**1.62)	1.39	(0.98–1.96)	**1.70**	**(1.03–2.80)**
50–64 years old	386	1.00	1.19	(0.89**–**1.60)	1.21	(0.88–1.67)	1.36	(0.93**–**2.00)
≥ 65 years old	409	1.00	1.16	(0.81–1.66)	**1.72**	**(1.21–2.45)**	**2.04**	**(1.44–2.89)**
Household members								
One person	233	1.00	1.59	(0.47–5.37)	1.87	(0.61**–**5.75)	2.37	(0.79**–**7.16)
Multiple persons(≥1)	1008	1.00	1.15	(0.97–1.38)	**1.38**	**(1.14–1.67)**	**1.56**	**(1.23–1.99)**
Region								
Metropolis	524	1.00	1.26	(0.96**–**1.63)	1.25	(0.94–1.66)	**1.70**	**(1.23–2.34)**
City	458	1.00	1.13	(0.84–1.50)	**1.53**	**(1.14–2.06)**	**1.73**	**(1.22–2.46)**
Rural area	259	1.00	1.00	(0.68–1.47)	1.35	(0.88–2.06)	1.21	(0.73–2.01)
Household Income								
Low	433	1.00	1.13	(0.79–1.61)	1.32	(0.91**–**1.90)	**1.87**	**(1.30–2.70)**
Medium-low	321	1.00	1.08	(0.78–1.48)	1.32	(0.87–1.98)	1.32	(0.87**–**1.98)
Medium-high	265	1.00	**1.76**	**(1.22–2.55)**	**2.02**	**(1.37–2.97)**	**2.04**	**(1.24–3.35)**
High	222	1.00	0.77	(0.54–1.11)	1.18	**(0.80**–1.74)	1.00	(0.56–1.78)
**Suicidal ideation (*n*** **= 479)**	**Case(n)**	**3(*n*** **= 108)**	**2(*n*** **= 117)**		**1(*n*** **= 128)**		**None(*n*** **= 126)**	
		Odds Ratio	Odds Ratio	95% CI^a^	Odds Ratio	95% CI^a^	Odds Ratio	95% CI
Generation								
20–29 years old	46	1.00	1.57	(0.51**–**4.83)	**4.22**	**(1.40–12.68)**	**4.24**	**(1.20–14.94)**
30–49 years old	118	1.00	1.06	(0.62**–**1.81)	**1.98**	**(1.15–3.40)**	2.03	(0.94–4.37)
50–64 years old	145	1.00	0.94	(0.59**–**1.51)	0.98	(0.59**–**1.64)	1.49	(0.85–2.61)
≥ 65 years old	170	1.00	1.48	(0.88–2.49)	**2.05**	**(1.22–3.44)**	**2.36**	**(1.43–3.92)**
Household members								
One person	101	1.00	0.68	(0.11**–**4.11)	1.51	(0.33**–**7.04)	1.63	(0.36**–**7.42)
Multiple persons(≥1)	378	1.00	1.15	(0.87–1.53)	**1.67**	**(1.24–2.24)**	**2.10**	**(1.48–2.96)**
Region								
Metropolis	197	1.00	1.08	(0.70**–**1.66)	1.39	(0.89**–**2.16)	**1.80**	**(1.10–2.94)**
City	179	1.00	1.31	(0.81**–**2.12)	**1.98**	**(1.24–3.18)**	**2.43**	**(1.44–4.11)**
Rural area	103	1.00	0.86	(0.47–1.59)	**2.07**	**(1.14–3.76)**	1.82	(0.90–3.72)
Household Income								
Low	185	1.00	1.00	(0.60**–**1.67)	1.49	(0.90–2.47)	1.55	(0.92–2.60)
Medium-low	133	1.00	1.16	(0.70**–**1.91)	1.42	(0.84–2.40)	**1.87**	**(1.04–3.36)**
Medium-high	90	1.00	**2.02**	**(1.02–4.01)**	**3.21**	**(1.63–6.32)**	**3.04**	**(1.34–6.91)**
High	71	1.00	0.66	(0.35–1.25)	1.19	(0.62–2.28)	2.06	(0.94–4.48)

^a^*CI* Confidence Interval

l

^c^Commensality is analyzed by Controlled variables includes household members, generation, Residential area, household income, educational attainment, occupation, chronic illnesses, smoking, drinking, except each subgroup variable

Women in the 20–29 age group were 4.22 times more likely to have suicidal ideation if they ate only one meal commensally (OR: 4.22, 95% CI: 1.40–12.68) and 4.24 times more likely if they ate all meals alone (OR: 4.24, 95% CI: 1.20–14.94). Women 65 years or older were 2.05 times more likely to have suicidal ideation if they ate one meal together (OR: 2.05, 95% CI: 1.22–3.44) and 2.36 times more likely if they ate alone (OR: 2.36, 95% CI: 1.43–3.92). Women who lived in cities were more likely to have suicidal ideation if they ate fewer meals commensally (one meal together, OR: 1.98, 95% CI: 1.24–3.18; no meals together, OR: 2.43, 95% CI: 1.44–4.11). Finally, women making medium-high incomes had significantly greater odds of suicidal ideation if they ate fewer meals commensally, whether that was two meals (OR: 2.02, 95% CI: 1.02–4.01), one meal (OR: 3.21, 95% CI: 1.63–6.32), or no meal together (OR: 3.04, 95% CI: 1.34–3.6.91)(Table 6).

## Discussion

Previous studies have demonstrated the importance of commensality for social interactions and intimate relationships [28]. Specifically, eating alone, without the benefits of commensality such as socializing and disclosure, was related to a greater likelihood of depression and suicidal ideation [29, 30]. Depression and suicidal ideation were analyzed together, because the former is highly correlated with suicidality (including suicidal ideation, suicidal plans, and suicidal attempts) [9, 30]. Our study focused on the benefits of commensality for promoting mental health. Numerous studies have linked not only physical health but also mental health to self-destructive behaviors such as suicide, suggesting the need to prevent these behaviors through an integrated approach [30–32]. We added to the existing literature by analyzing the strength of the relationship between commensality and mental health for various subgroups, using detailed socio-economic data on Korean adults.

The results showed that both men and women who ate meals less frequently with others were more likely to be depressed. This result differs from that of previous studies, in which commensality had a strong association with depression only among men [33]. Also, we found that commensality was significantly associated with depression and suicidal ideation for the 20–29 year old age group, in contrast with previous studies that only found these associations among older adults [24, 34–36]. For early adults, commensality provides emotional stability and positively affects mental health [6, 7]. Increased pressure in the academic, marriage, and employment realms has forced young adults to delay getting married and live alone for a longer period, which causes them to have individualistic values and decreases their social exchanges with others [37].

This study also demonstrated that lower socio-economic levels [24, 35, 38, 39], including lower income levels and education attainment, and poorer physical health, such as the present of a chronic disease [40] or smoking [14], have a higher association between eating alone and depression and suicidal ideation.

Similar to research that found that commensality with family members has a positive effect on mental health [24, 33], this study also found that people in multi-person households who ate meals alone were more likely to be depressed and have suicidal ideation. Owing to the small sample size and the fact that the proportion of single-person households was only 10%, the relationship between commensality and mental health was insignificant among single-person households. Because the population of single-person households is increasing in Korea [41], further research is needed to explore the effect of eating alone for those who live alone.

Because many unmarried and young men have moved to cities and bereaved and old women have stayed in rural communities in Korea, there are residential and cultural differences in mental health. Prior research has shown that those living in rural areas with low income levels tended to have increased levels of depression [42]. This study found that men are more likely to be depressed if they are living in a smaller population area in a rural area, followed by cities and metropolises. Moreover, the odds of suicidal ideation was higher in cities, followed by rural areas and metropolises. Women were, on the other hand, more likely to be depressed and higher suicidal ideation in cities unlike previous studies showed that women in rural areas were significantly more depressed [37, 42].

A major limitation of this study is its cross-sectional nature. The lack of longitudinal data meant we cannot comment on the causality of commensality; we do not know if eating together directly improved mental health, or if depression and suicidal ideation conversely caused participants to seek out company at mealtimes. In particular, we did not exclude or reclassify individuals who were previously diagnosed with depression, because we could not determine whether depression influences the likelihood of commensality or vice versa. We also did not account for the possibility that individuals may want to eat alone, and that such a choice may be positive depending on their own preferences and health. Given the wide range of factors affecting dietary changes in modern society, future studies should carefully separate the various causes of diet-related behaviors to clarify any links between commensality and mental health. And almost 20% participants in the datasets were excluded as missing values, non-specified or no answers in the self-reported health survey, particularly about diagnosis of depression. Considering the possibility of losing those with depression did not desire to answer, the results should be carefully interpreted.

Nevertheless, our study has important strengths. We considered important covariates (e.g., socioeconomic factors, chronic conditions) in our analysis of commensality, identifying statistically significant associations between eating habits and mental health that differed depending on household size and residence type (urban vs. rural). Notably, we were able to compare adults living alone but still ate commensally with those who lived with others and ate commensally. This analysis allowed us to focus specifically on the mental-health effects of eating alone that were distinct from cohabitation. Our findings should present directions for further research on the link between households and depression or suicide. In addition, through our inclusion of young and middle-aged adults, we expanded the applicability of the results compared with previous studies that focused only on the elderly. Finally, we examined social structure characteristics (e.g., income level) that may modulate the association between eating alone and depression/suicidal ideation in adults. Understanding these interactions could provide better policy directions for addressing mental health problems in a population.

## Conclusions

In conclusion, this study provided evidence that commensality was important for mental health. We demonstrated the need to consider individual characteristics and social networks when examining this link. Thus, future studies should include these factors when exploring further questions on commensality, for instance whether an individual desires eating together or wishes to avoid it, and whether the causes underlying solitary eating differ in single vs. multi-person households. Overall, given that our data suggest social isolation from eating alone could deteriorate both physical and mental health, social workers, educators and also policy developers to be aware of the importance of eating together and develop and to promote programs that encourage commensality. Our results are valuable as a basic resource for panel data analysis or a nested case-control study to identify sequential and casual relationships between commensality and mental health.

## Data Availability

Not applicable.
